# The Physiology of the Nerves of the Uterus and Its Appendages

**Published:** 1846-04

**Authors:** 


### The Physiology of the Nerves of the Uterus and its Appendages. By *Joseph Swan*. London, 1846. Pp. 31.

This small pamphlet owes its appearance, we presume, to the controversy which has for some time existed respecting the state of the nerves distributed to the uterus during gestation, and which has attracted special attention during the last year. We need not remind our readers of the views entertained by Dr. Robert Lee respecting the enlargement of the nerves and their ganglia in the gravid uterus, as they have been so often brought before the notice of the profession. In an interesting paper read before the Royal Society, in the Session preceding the present, and to which subsequently the Council have awarded one of the gold medals of the Society, Mr. Beck contends that the uterine nerves *do not experience any increase in size during pregnancy*, an assertion resting on some most beautiful and successful dissections of the hypogastric and sacral plexuses. As this paper is not yet published in the Philosophical Transactions, we extract the following brief notice contained in the printed "Proceedings."

"On the Nerves of the Uterus." By Thomas S. Beck, Esq. Communicated by Sir Benjamin Brodie, Bart. F.R.S.

"The object of the author in this communication is to record the results of his dissections of the nerves of the uterus, both in the unimpregnated and gravid states, with a view to determine if any changes are observable in them in these two conditions. He enters minutely into the anatomical details of the formation of the great splanchnic nerve, the composition of the semilunar ganglion, and the distribution of the branches proceeding from it to the different abdominal viscera. His conclusions are, that while the ovaria derive their nerves from the renal, the fallopian tubes from the hypogastric, and the bladder, rectum and vagina from the pelvic plexus, the nerves supplying the uterus are continuations of the hypogastric plexus, and that they undergo, during pregnancy, no further change, either in size or position, except that which is the simple consequence of the enlargement of the organ over which they are distributed, and that they undergo no other change during a second pregnancy. He thinks it probable, moreover, that the vessels of the uterus do not decrease in size after parturition, but are only contracted in their cavity. He notices several points relating to these subjects, which are still open to further investigation. The paper is accompanied by highly finished drawings of the appearance of the dissected parts."—(*Proceedings of the Royal Society,* 1845, P. 562.)

That this investigation is an important one, must be inferred by the sanction it has received from some most competent judges; but it is due to a very diligent cultivator of this branch of anatomy, Dr. Lee, to state that the correctness of his researches has also been admitted by many distinguished observers, and further, as appears from the following communication made to the Royal Society subsequently to the reading of Mr. Beck's paper, that that gentleman remains convinced of the truth of his original conclusions.

"Supplement to a Paper 'On the Nervous Ganglia of the Uterus.' By Robert Lee, M.D., F.R.S., Fellow of the Royal College of Physicians.

"The author is confirmed in his views regarding the arrangement of the nervous filaments distributed to the uterus, as described in his papers printed in the Philosophical Transactions for 1841 and 1842, by his recent dissection of a gravid uterus at the full period, and which he considers as demonstrative of the accuracy of all the statements which are contained in those communications."—(*L. c.* p. 566.)

As the point in dispute may be more advantageously considered when Mr. Beck's communication shall have been published, we proceed to notice Mr. Swan's brochure. The author states that the principal facts now re-published, and the leading opinions founded on them, originally appeared several years ago, but that he has been induced again to present them to public notice, because it appeared that they required a fuller explanation.

One principal object with Mr. Swan, is to show that increased action may take place in a part without its nerves being necessarily enlarged. He observes:—

"When portions of the body and their particular faculties are separately noticed, it may appear as if changes of function could not be produced without some alteration of structure; and although this may be the case in many instances, yet changes will be found for which neither structure nor ordinary faculties will account, and it is only on an extensive survey of the animal economy that coincidences will occur to remove some of the obscurities, and further suggest that there may exist modifications of faculties which may be prominent in some animals and seldom apparent in others, and that these may be enhanced by altered qualities of the more immaterial or spiritual part of the nervous system. In this manner the apparent leading peculiarities of the faculties of one animal may be brought to produce an additional or extraordinary power in others for accomplishing some particular object. The dominant passions and faculties of the brain and instinct of some animals can, on an emergency, be roused into action in others, to which they are in the same very high degree almost entirely strangers. They are excited either by external objects, or by the activity of some organs, as the stomach, the ovaries, or the testes. In some, a process bearing a slight analogy to reproduction is carried on in various parts, as in the growth of deciduous horns, or the permanent growth of the front teeth of rodent animals; also in the growth of hair."—(*The Physiology of the Nerves of the Uterus*, p. 6.)

This position is supported by several instances, in which "the changes in the nervous system do not appear to depend on an altered structure, but on the altered qualities of the immaterial or spiritual parts." Thus, in pregnancy, these qualities are exalted; and, previously to the commencement of parturition, the altered quality of the nerves of the lower part of the uterus, the vagina, and of the external parts, by which their sensibility is exalted, becomes apparent. Again—

"If much power is required in a part not actuated by the will, it is supplied by large sentient nerves, which are so combined with the motive at particular points, as to be able to call muscles into action; this is done in the face, and particularly on the surface of the body, in which the cutaneous muscle exists." "By the acquisition of enhanced, or new qualities, small and safe means are sufficiently substituted for extraordinary occasions, rather than larger provisions, which might be prejudicial in the ordinary functions of the animal: thus very small and apparently insignificant nerves are used for causing very powerful effects, by combining them with parts capable of undergoing irritable changes, and thus imparting to them sufficient exciting qualities at the time the expulsion of the fœtus becomes necessary." (*L. c.* p. 11.)

The conclusion to be drawn from all these arguments is, that as organs can assume an unwonted degree of activity, in virtue of the exalted qualities of their nerves, there is no just reason why the nerves of the uterus should be enlarged during pregnancy. Although we conceive that no action ever occurs without a corresponding change in the organ employed, yet this may often consist of a merely temporary alteration, such as an increased flow of blood; so that we concur in the general truth of Mr. Swan's inference, though on somewhat different and more restricted grounds.

In proceeding to consider the particular faculties of the nerves employed in conception, gestation, and parturition, the author endeavours to establish an important distinction between the power of the sympathetic and spinal nerves in exciting muscular action; the impulses of the former are just sufficiently strong to excite the involuntary contraction of the muscular coat of the viscera, "but when any part is so much irritated as to become painful, the impression on the sympathetic is conveyed to the sensory indirectly, by communications with the spinal marrow by the spinal nerves, and voluntary muscles are thus brought to act with them on some very urgent occasions." In this manner, the "gentle perceptive quality" of the sympathetic is therefore well adapted for supplying the uterus which is to contain the growing fœtus; but to excite the violent expulsive actions of the abdominal muscles in parturition, spinal nerves are requisite, as they alone are extremely sensitive, and "at once convey impressions to the sensory nerves, and rouse the voluntary powers to free the parts they supply from compressing and irritating causes."—(*L. c.*, p. 15.)

The mode in which the ovary, the uterus, and the vagina are supplied with nerves, corresponds with the physiological principles here enunciated; for, whilst the germ-preparing organ, the ovarium, and the part of the uterus particularly engaged in nourishing and developing the embryo, namely, the fundus, are exclusively supplied by the great sympathetic (by means of the spermatic and hypogastric plexuses), the lower part of the uterus and the vagina, which are specially concerned in exciting, by their augmented sensibility, the expulsive actions, derive their supply partly from the sympathetic and partly from the sacral nerves. It is a remarkable and interesting fact, that the nerves of the unimpregnated uterus are very small, and present a striking contrast when compared with those of the bladder, vagina, and rectum.

The author describes a particular fibrous expansion connected with the round ligaments, and placed between a considerable portion of the muscular parietes of the uterus and the peritoneum, which increases very much in the last period of pregnancy; its use is mechanical. Mr. Swan seems, in the following passage, to imply that this texture might be mistaken for a nervous structure, especially if the colour of the parts had been previously removed, as by spirits of wine. "The nearer the full period of gestation, the more developed is this structure, and then its identity with nerve cannot be entertained by the most inexperienced; there is a greater resemblance at an earlier period, and particularly after it has been blanched in fluids. It would have been contrary to the economy of the nervous system, and particularly when the brain is very large, for a mass of nerve equal to this fascia to have existed, and it is not possible to conceive that the term of utero-gestation would ever have been completed had it partaken of the nervous function, and been stimulated by the present copious supply of blood. Such a power would have been incompatible with its connection with any organ so irritable as the uterus, when it contained a living embryo."—(*L. c.*, p. 20.)

It would not be doing justice to the author in noticing a pamphlet like this, in which, for the sake of brevity, everything is condensed into a small compass, if we did not present the general conclusions at which he has arrived in his own words; we therefore extract the following summary.

"It may not be uninteresting to recapitulate the leading facts set forth in the preceding pages, for showing that there are various powers implanted in the animal body for its preservation, and capable of being called into activity on proper occasions; that variations of faculties and qualities more or less prominent, or entirely quiescent, form these means, throughout the animal creation; that provisions have been thus made for accommodating one part of the body to another, that useless mechanism might be avoided, when it could not be diminished or removed, and it would remain as an incumbrance after the completion of any special process; also for adapting the animate portion of the creation to the variations of the inanimate, through which the animate is to be more or less sustained or destroyed. It has been stated that there need not be altered structure when there are altered functions; that there are natural processes, neither requiring nor producing more than a very slight local excitement; that natural processes in the mature body of some animals are resembled by the growth of the embryo; that there are variations of natural processes which are healthy in one animal and morbid in another: that there are restorative processes analogous to the natural healthy ones; that there are modifications of excited faculties in reproduction which are healthy in some animals and morbid in others; that parturition, although a healthy process, approaches very near to the morbid; that there are increased natural motive powers resembling tetanus; that there is a general altered quality of the nervous essence, and particularly after conception, for expediting the maturity of the fœtus, and producing a higher excitement for the completion of parturition; that there is a local power of excitement produced in the nerves by the excited condition of the lower part of the uterus and vagina: that the local actions are modified by the peculiar qualities of different nerves, and are further changed by combinations between themselves; that the sympathetic is well adapted for the functions of the ovary, also for the increase of the uterus and the nourishment of the fœtus, but is not sufficient for exciting full expulsive powers; that the branches from the plexus of the conjoined hypogastric and sacral nerves at the lower part of the uterus and vagina are proper for receiving irritation at the full maturity of the fœtus, or on the accession of any morbid cause, and are then capable of exciting tetanic action; that the sentient nerves of the external parts are proper for rousing the voluntary muscles to expel the fœtus; that the fibrous structure connected with the round ligaments and placed between the peritoneal and muscular coats has entirely mechanical powers for preserving the attachment of the peritoneum to its proper place after the contraction of the uterus; that the plexus of conjoined hypogastric and sacral nerves is not proper to the uterus, but to the bladder and rectum; that in uniparous animals the nerves and blood-vessels are severally arranged for retarding the growth of the uterus and fœtus; that in multiparous animals every part of the uterus, except the very lowest, is fully and freely adapted, by the arrangement of the blood-vessels and nerves, for expediting the maturity of the fœtuses, and that the portions only containing the fœtuses can receive sympathetic nerves; that if there be any enlargement of the uterine nerves, it is very slight, and that such a power would not have been proper for securing the gentle action of the uterus during gestation, if it could have promoted the violent one in parturition."—P. 28-30.

Before concluding this notice, it is requisite to state that, in many parts of his essay, the author has announced as settled facts, what are merely speculations, and speculations, moreover, many of which we believe to be opposed to the laws of organic and animal life. For example, in alluding to the arrangement of the uterine vessels, he affirms that "as arterial blood might be more stimulating than is consistent with the inactive life of the fœtus, it is generally furnished by the veins which are accompanied by nerves for regulating the quantity, and giving a modified and requisite impetus, and in this respect there is an approach to the supply by the portal vein of the liver, and its accompanying nerves." In answer to this hypothesis, it may be remarked, that no physiologist in the present day imagines that there is any direct communication between the maternal and fœtal vessels, by which the arterial blood of the former is conveyed to the latter; on the contrary, every advance in this direction, and more particularly we would refer to the researches of Weber, Sharpey, Dalrymple, and Goodsir, shows that it is a deposit or secretion from the uterine blood which is conveyed to the fœtus; not that there is any special reason why arterial blood should not circulate in the umbilical vein, a circumstance which does actually occur, among many other examples, in the chick.

Again, we conceive that the author has exaggerated the influence of the nerves over the formation of the ovum and its subsequent development. Nor do we think it is at all proved that, in parturition, the abdominal muscles are excited essentially by the branches of the spinal nerves distributed to the lower part of the uterus, the vagina, and the labia; it is much more in keeping with what is observed in convulsion induced by irritation of the mucous surface of the intestine, to infer on the contrary, that the branches of the sympathetic distributed to the uterus are primarily affected at the approach of parturition, and that they excite the spinal action. Without desiring in any degree to detract from the merits of the author, we cannot withhold the expression of our regret that he should, on such a slender foundation, have so largely indulged in that theorising spirit, which has proved so great an obstacle to the progress of true physiology, and which of late years, has fallen into deserved disrepute.

